# The Reciprocal Relationship Between Cell Adhesion Molecules and Reactive Oxygen Species

**DOI:** 10.3390/cells14141098

**Published:** 2025-07-17

**Authors:** Muayad Al-Hadi, Alexander G. Nikonenko, Vladimir Sytnyk

**Affiliations:** 1 School of Biotechnology and Biomolecular Sciences, The University of New South Wales, Sydney, NSW 2052, Australia; m.al-hadi@unsw.edu.au; 2 Department of Cytology, Bogomoletz Institute of Physiology, 01024 Kyiv, Ukraine; alexander.nikonenko@ukr.net

**Keywords:** cell adhesion molecule, integrin, cadherin, immunoglobulin superfamily, selectin, reactive oxygen species, cell death

## Abstract

Cell adhesion molecules (CAMs) are cell-surface-localized proteins mediating interactions of cells with other cells and the extracellular matrix. CAMs influence cell behavior and survival by inducing various intracellular signaling cascades that regulate diverse cellular processes including cytoskeleton remodeling and gene expression. Here, we review the evidence demonstrating that the levels, subcellular distribution, and binding affinities of CAMs of several major families including integrins, cadherins, immunoglobulin superfamily, and selectins are regulated by intracellularly generated or extracellular reactive oxygen species (ROS). Remarkably, CAMs themselves induce ROS production in response to binding to their ligands by activating lipoxygenases or NADPH oxidases or influencing ROS generation in mitochondria. CAM-dependent ROS production is essential for CAM-mediated cell adhesion and CAM-dependent intracellular signaling. Importantly, CAMs also protect cells from the ROS-induced cell death by stimulating the synthesis of antioxidants and suppressing the cell death signaling. A better understanding of the role ROS play in controlling CAM functions and mechanisms of this control may pave the way to modulating the functions of CAMs in various disorders associated with abnormal cell adhesion.

## 1. Introduction

### 1.1. Major Families of CAMs

Cell adhesion molecules (CAMs) are a group of glycoproteins that accumulate at the cell surface and mediate both cell–cell adhesion as well as cell–extracellular matrix (ECM) interactions. Here, we focus on the major families of CAMs including integrins, cadherins, the immunoglobulin superfamily (IgSF), and selectins. Structural features of these proteins were reviewed previously [1,2,3] and are introduced here only briefly. All CAMs are characterized by the presence of a large extracellular domain that mediates adhesion. Integrins are made out of α- and β-subunits forming αβ dimers. They mediate adhesion to the extracellular matrix proteins, but can also interact with other CAMs, such as some IgSF members. The extracellular domain in cadherins is characterized by the presence of cadherin modules. It can bind to the extracellular domain of another cadherin molecule, thereby mediating homophilic adhesion. Cadherins form adhesive bonds only in the presence of calcium ions. The extracellular domain of IgSF members is characterized by the presence of one or more immunoglobulin-like (Ig) modules, but can also contain other domains, most often fibronectin type III modules. IgSF members mediate homophilic adhesion and can also heterophilically interact with other cell surface receptors and CAMs, such as integrins. Selectins share some properties with lectins and bind to sugar moieties. Most CAMs are transmembrane proteins with a short transmembrane domain and a cytoplasmic tail. However, some CAMs are anchored to the plasma membrane by the glycosylphosphatidylinositol anchor and lack transmembrane and intracellular domains [4]. Additionally, cells express CAMs that do not belong to the major families, such as neurexins and neuroligins, which have been shown to play important roles in the nervous system [1]. Intracellular domains of CAMs interact with multiple cytosolic proteins including cytoskeletal linker proteins and various enzymes. In addition to mediating adhesion, CAMs induce various intracellular signaling cascades regulating cytoskeleton remodeling, gene expression, protein trafficking, and other cellular processes [5,6,7,8].

### 1.2. Reactive Oxygen Species

‘Reactive oxygen species’ (ROS) is a collective term describing the chemical species formed upon the incomplete reduction of oxygen. Types of ROS and mechanisms of their production have been extensively reviewed previously [9] and are discussed here only briefly. Some of the most common ROS are the superoxide anion (O_2_^−^), hydrogen peroxide (H_2_O_2_), and the hydroxyl radical (HO•). These inorganic molecules are rapidly diffusible, highly reactive, and tightly controlled by scavenger systems. ROS can be generated as intermediates in the oxygen reduction pathway in mitochondria and are thought to mediate the toxicity of oxygen because of their chemical reactivity [10]. In addition, ROS are produced by enzymes outside the mitochondrial respiration system, for example by lipoxygenases (Lox), cytosolic enzymes binding to lipid membranes and dioxygenating unsaturated fatty acids [11]. At the plasma membrane, ROS are generated by NADPH oxidases (Nox). These transmembrane proteins transport electrons from cytosolic NADPH, through flavin adenine dinucleotide, across the plasma membrane to molecular oxygen to generate superoxide [12]. Among ROS, hydrogen peroxide is less reactive, has a longer lifetime, and can oxidize cysteine residues of redox-sensitive proteins to modify their activity. Because of that, ROS, and hydrogen peroxide in particular, play significant roles as critical regulators of various intracellular signaling pathways [10].

## 2. CAMs Influence Cellular Levels of ROS

### 2.1. Integrins

Early studies showed that artificial and natural ligands of integrins induce an increase in the cellular levels of hydrogen peroxide in fibroblasts. For example, in rabbit synovial fibroblasts, the ligation of α5β1 integrin using an adhesion-perturbing monoclonal antibody against α5β1 integrin leads to the production of hydrogen peroxide [13]. The attachment of resuspended NIH-3T3 fibroblasts to plastic dishes coated with an extracellular matrix protein fibronectin brings an up-to-10-fold increase in levels of cytoplasmic hydrogen peroxide starting at 15 min, peaking at 45 min and then slowly decreasing [14].

**Figure 1:**
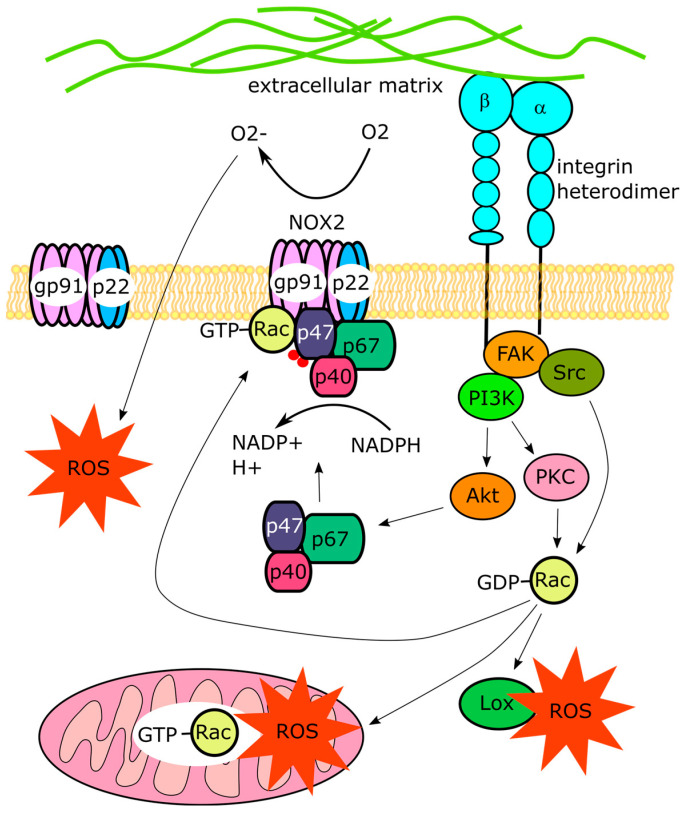
Mechanisms of the integrin-induced increase in cellular ROS levels. Binding of integrins to the extracellular matrix ligands and integrin clustering lead to activation of downstream signaling kinases including FAK, Src, PI3K, and PKC, which activate guanine nucleotide exchange factors promoting conversion of inactive GDP-Rac to active GTP-Rac. The active form of Rac is an obligatory subunit of Nox1 and 2 complex regulated by integrins (Nox2 complex is depicted). The assembly of this complex is also promoted by Akt, which phosphorylates its organizer p47phox (phosphorylation is depicted as red dots), resulting in the active Nox2 complex assembly. Superoxide generated by Nox2 diffuses into the cytosol, leading to an increase in cellular ROS levels. Active Rac also induces cytosolic ROS production by Lox and mitochondria, shown at the bottom.

Nox, Lox, and mitochondria contribute to the integrin-induced ROS production in fibroblasts (Figure 1). For example, in resuspended fibroblasts incubated with antibodies against α5 integrins, integrin-induced hydrogen peroxide generation is reduced by the inhibitors of Lox and partially of Nox [14]. ROS production in NIH3T3 fibroblasts triggered by the attachment to fibronectin occurs in phases with mitochondria contributing to hydrogen peroxide generation at the early phase and Lox generating most of the hydrogen peroxide at the later phase of cell adhesion and spreading [15]. The α5 integrin-induced activation of hydrogen peroxide production in rabbit synovial fibroblasts is accompanied by the depolarization of the mitochondrial membrane being blocked by rotenone, antimycin A, and KCN, the inhibitors of the function of mitochondrial respiratory chain complexes 1, 3, and 4, respectively [16]. It should be noted that rotenone and antimycin A themselves induce ROS formation [17,18]. Rotenone induces a rise in ROS levels in the mitochondrial matrix and reduces mitochondrial ROS in the cytosol whereas antimycin A induces cytosolic mitochondrial ROS generation and inhibits ROS production in the mitochondrial matrix [19]. Their inhibitory effect on integrin-dependent ROS production suggests a complex relationship between different sources of ROS.

The integrin-induced production of ROS by Nox, Lox, and mitochondria depends on the activation of a small GTPase Ras-related C3 botulinum toxin substrate (Rac) [20], a member of the Rho GTPase family that includes Rho, Rac, and Cdc42. Ligand-bound integrins recruit and promote the activation of focal adhesion kinase (FAK) and Src kinase, thereby initiating a signaling cascade converging on the guanine nucleotide exchange factors, such as β-PIX or DOCK1, which promote Rac activation (reviewed in [21]). Activated Rac is an obligatory subunit of the active Nox complex [22,23]. Rac-1 is also imported into mitochondria, where its activated form promotes the generation of hydrogen peroxide [24,25]. In rabbit synovial fibroblasts, the α5β1-integrin-antibody-induced generation of hydrogen peroxide requires the activation of Rac-1 but does not depend on RhoA or Cdc42 [13]. The activation of α5 integrins in these cells leads to a transient increase in levels of active GTP-bound Rac that precedes the rise in hydrogen peroxide levels. It is required for the enhanced production of hydrogen peroxide, indicating that integrin ligation triggers Rac activation upstream of ROS formation [16]. In human BJ fibroblasts, the extracellular matrix protein CCN1 binds to α6β1 integrins and induces Nox1-dependent ROS generation and cellular senescence. This effect is blocked in cells with depleted Rac-1 [26]. In primary human fibroblasts, CCN1 binds to integrins αvβ5, α6β1, and syndecan-4, triggering ROS generation, which is blocked by antibodies against αvβ5 or α6 integrins but not by antibodies against αvβ3 integrins. This CCN1-induced integrin-dependent ROS production is also blocked in cells with depleted Rac-1 or treated with the Rac-1 inhibitor. Interestingly, the inhibitor of arachidonate 5-lipoxygenase (5-Lox) and rotenone, but not the inhibitor of Nox, completely blocks the CCN1-induced ROS generation in these cells [27].

The integrin-dependent regulation of ROS production is not limited to fibroblasts but occurs in a variety of other cell types. For example, the amyloid β peptide elevates ROS levels in astrocytes via the β1 integrin-dependent activation of phosphatidylinositol 3-kinase (PI3K) signaling, leading to the activation of protein kinase C (PKC) and PKC-dependent activation of Rac-1 and Nox [28]. In colic adenocarcinoma Caco-2 cells, the binding of α2β1 integrins to collagen induces superoxide production via the activation of Nox [29]. In rat aortic smooth muscle cells, the activation of integrins using tetrapeptide Arg–Gly–Asp–Ser (RGDS) also causes an increase in hydrogen peroxide levels [30]. The RGDS-induced hydrogen peroxide production is suppressed by transfecting cells with siRNA, reducing the levels of NADPH oxidase 4 (Nox4) and its regulator polymerase δ-interacting protein 2 (Poldip2) [30]. In non-malignant mammary epithelial cells, the overexpression of the “gain-of-function” mutant of β1 integrin, an increase in the density of fibronectin-bound integrins, or the acute activation of integrins using manganese enhances mitochondrial ROS production in part through regulating the activity of the Na^+^/H^+^ exchanger SLC9A1 responsible for the efflux H^+^ from the cytoplasm and control of mitochondrial morphology through changes in intracellular pH [31]. In platelets, the agonist-dependent activation of αIIbβ3 integrins under sheer stress triggers hydrogen peroxide production via NADPH oxidase 2 (Nox2) activation. This activation is triggered by the phosphorylation of the organizer of the Nox2 complex p47phox at S^304^ and S^328^, resulting in the assembly of the active Nox2 complex. The integrin-dependent phosphorylation of p47phox relies on the binding of the intracellular domain of β3 integrin to Gα13 and activation of the PI3K-Akt pathway while neither mitogen-activated protein kinase (MAPK) nor PKC are required [32]. In cardiomyoblast H9c2 cells, the extracellular matrix protein CCN1 induces an increase in ROS levels and promotes Fas ligand-induced apoptosis by binding to α6β1 integrin. This effect is reduced by inhibitors of 5-Lox and Nox [33].

The production of ROS under physiological conditions is highly compartmentalized [34], and integrins are likely to be involved in the spatial control of ROS production. For example, in migrating endothelial cells, integrins accumulate at the leading edge [35,36] where Nox2 also accumulates and where the formation of ROS is spatially and temporally controlled to promote directional cell migration [37]. Integrins promote Nox-dependent hydrogen peroxide production not only at the plasma membrane but also in intracellular organelles. In articular chondrocytes, α5β1 integrins can be activated by a fibronectin fragment containing the RGD integrin attachment site and DRVPHSRN synergy site that together promote the opening of α5β1 to the active conformation. The binding of α5β1 integrin to this fibronectin fragment leads to the internalization of the ligand–integrin complex and hydrogen peroxide generation starting at the plasma membrane and continuing later in Nox2-containing early endosomes or “redoxosomes” [38]. The organizer of the Nox2 complex p47phox is phosphorylated in response to the fibronectin fragment, suggesting that α5β1 integrins activate Nox2 by inducing the phosphorylation of its organizer. Interestingly, Nox4 is not activated by α5β1 integrins and is not recruited to endosomes in chondrocytes [38].

Integrins are also implicated in the inhibition of ROS production. The activation of integrins in human neutrophils adhering to fibronectin results in an initially delayed, but subsequently enhanced, generation of ROS in response to a variety of soluble activators. The initial suppression represents a protective mechanism to prevent inappropriate tissue damage and is mediated by integrins suppressing ROS formation by inhibiting Rac-2. The activation of integrins enhances tyrosine phosphatase activity, leading to the dephosphorylation of the regulatory Tyr174 in a guanine nucleotide exchange factor, Vav1, resulting in reduced Vav1 and Rac-2 activity [39]. In mesangial cells, the lack of α1β1 integrins results in increased ROS levels [40]. The loss of α1β1 integrins induces a constitutively upregulated Rac-1 activity due to the enhanced phosphorylation of the epidermal growth factor receptor (EGFR) and consequent activation of Vav2. Activated Rac-1 is translocated to the plasma membrane, resulting in excessive ROS production [41]. In mouse embryonic fibroblasts, αvβ3 but not α5β1 integrins reduce ROS levels and suppress the platelet-derived growth-factor-induced ROS production at focal adhesion sites via the activation of RhoA [42].

Together, current evidence suggests that integrin activation can either enhance or inhibit ROS generation in a cell- and tissue-specific manner.

### 2.2. Cadherins

Fat1 is an atypical cadherin with a very large ectodomain, comprising thirty-four cadherin repeats, five epidermal growth factor (EGF)-like repeats, and one laminin G-like domain. In *Drosophila*, the intracellular domain of Fat1 is cleaved and its soluble fragment is imported into mitochondria, where it binds to NADH dehydrogenase ubiquinone flavoprotein 2 (Ndufv2), a core component of complex I. The loss of Fat1 reduces complex I activity and elevates ROS levels [43]. In mammalian vascular smooth muscle cells, fragments of Fat1 accumulate in mitochondria, where its intracellular domain interacts with multiple mitochondrial proteins limiting complex I and II activities [44].

### 2.3. IgSF Members

Several members of the IgSF family have been shown to be implicated in regulating cellular ROS levels. In the murine microvascular endothelial MS-1 cells, the cytoplasmic tail of intercellular cell adhesion molecule 1 (ICAM-1) increases the generation of the tripeptide glutathione, the most abundant intracellular antioxidant formed by the rate-limiting heterodimeric enzyme glutamate cysteine ligase (GCL). The cytoplasmic tail of ICAM-1 increases glutathione levels by stimulating GCL via Nox4-dependent ROS production and the activation of PI3K signaling [45]. In neurons, the myelin basic protein cleaves the L1 cell adhesion molecule (L1CAM), generating the transmembrane cleavage product of L1CAM, called L1-70, which is transferred to endosomes, then released from them into the cytosol and imported into mitochondria. In these organelles, L1-70 increases complex I activity and the generation of the mitochondrial membrane potential by binding to the complex I subunit NDUFV2. Furthermore, the L1CAM fragment promotes mitochondrial fusion, fission, motility, and trafficking by binding to the mitochondrial Rho GTPase 1 and dynamin-related protein 1 [46,47,48]. Interestingly, two other members of this family, neural cell adhesion molecule (NCAM) and close homologue of L1 (CHL1) are not involved in regulating the mitochondrial function [46]. Mutations in L1CAM cause L1 syndrome in humans. In neurons from mice expressing L1CAM with the L1 syndrome causing missense mutation D201N, mitochondrial complex I activity and ATP levels are normal while the mitochondrial membrane potential and ROS levels are higher with respect to wild-type neurons [47]. These data suggest that L1CAM mutations affect neuronal survival by increasing ROS levels. Transmembrane and immunoglobulin domain-containing protein 1 (TMIGD1) is an IgSF CAM predominantly expressed by epithelial cells of the intestine and kidney. The PDZ domain-binding motif within the TMIGD1 intracellular domain interacts with synaptojanin 2-binding protein, which recruits TMIGD1 to mitochondria in low-density cultures of human renal epithelial HK-2 cells derived from proximal tubules, while TMIGD1 redistributes to cell-to-cell contacts at higher cell densities [49]. In mesothelial cells overexpressing TMIGD1, the 500 μM hydrogen-peroxide-induced increase in ROS levels, mitochondrial damage, and cell death are reduced, indicating that TMIGD1 protects cells from oxidative injury [50].

## 3. CAM-Induced ROS-Production Regulates Intracellular Signaling, Transcription, and Cytoskeleton Remodeling

ROS produced in response to CAM activation play an important role as signaling molecules transducing signals from the plasma membrane into the cell, where they induce a variety of effects including the remodeling of the cytoskeleton and transcription activation in the nucleus. These effects have been particularly well studied for integrins. For example, the attachment of NIH-3T3 fibroblasts leads to the oxidation and inhibition of acid phosphatase 1, also known as LMW-PTP, which dephosphorylates and inactivates FAK. The inhibition of LMW-PTP facilitates the formation of focal adhesion sites by increasing FAK activation [14]. The activity of LMW-PTP then slowly recovers, this effect being dependent on the intracellular reduced glutathione [14]. The activation of integrins during the attachment of NIH-3T3 fibroblasts to fibronectin coated plates also results in oxidation and the activation of Src kinase, leading to the association of Src with FAK and the activation of FAK [51]. These effects are inhibited in cells treated with the Lox-specific inhibitor, indicating that they are induced by the Lox-produced hydrogen peroxide [51]. In chondrocytes, α5β1 activation also results in oxidation and the activation of Src; however, in this case, it is induced by hydrogen peroxide produced by Nox2 [38]. In primary normal human skin fibroblasts, CCN1 promotes the tumor necrosis factor α (TNFα)-induced activation of c-Jun N-terminal protein kinase (JNK) by binding to α6β1 integrins, and this effect is abolished in cells treated with the ROS scavengers, rotenone, or Lox inhibitor but is not affected by the knock-down of Nox1 [27]. In rabbit synovial fibroblasts, the integrin-dependent generation of ROS triggers the rapid nuclear translocation of nuclear factor kappa B (NF-κB), a reduction/oxidation (redox)-sensitive transcription factor, resulting in the NF-κB-dependent transcriptional regulation of interleukin-1α, which, in an autocrine manner, induces collagenase-1 gene expression [13]. In human BJ fibroblasts, CCN1 activates extracellular-signal-regulated kinases (ERKs) and p38MAPK by binding to α6β1 integrins, and this effect is abolished by the Nox inhibitor. Activated p38MAPK triggers p16^INK4a^ induction and cellular senescence [26]. Integrins induce the ligand-independent and potentiate ligand-dependent activation of EGFR [3]. Both effects are required for rescuing adherent cells from a particular type of apoptosis called anoikis that is triggered by the accumulation of a proapoptotic protein Bim being degraded when integrins are activated in attached cells. In human ECV304 cells, the ROS scavenger N-acetyl-cysteine and 5-Lox inhibitor, nordihydroguaiaretic acid, block the ligand-independent activation of EGFR by integrins, reduce the EGF-dependent activation of EGFR, and inhibit both the integrin-dependent degradation of Bim as well as integrin- and EGF-dependent protection against anoikis [20]. In rat aortic smooth muscle cells, integrin activation caused by cell attachment to the substrate or RGDS peptides results in the oxidation of F-actin, but not of G-actin. Notably, this F-actin oxidation is suppressed by PEG-catalase depleting intracellular hydrogen peroxide, indicating that F-actin is oxidized by hydrogen peroxide generated in response to integrin activation [30]. The oxidation of F-actin is required for efficient cell migration, as evidenced by the impaired migration of cells with oxidation-resistant β-actin. This effect is linked to the maturation of focal adhesions since the inhibition of F-actin oxidation by the knock-down of Poldip2 or Nox4 results in the reduced recruitment of zyxin, the marker of mature focal adhesion [30]. Altogether, previous research has indicated that ROS production is an integral part of the intracellular signaling activated by integrins. However, the role ROS play in intracellular signaling by other CAMs requires further investigation.

## 4. CAMs Protect Cells from ROS-Induced Cell Damage and Death

### 4.1. Integrins

Although integrins promote ROS generation, they are also involved in mechanisms protecting cells from ROS-triggered cell death. Endothelial cells lining blood vessels are particularly exposed to ROS present in the bloodstream, especially to the superoxide anion generated by NADPH oxidases and xanthine oxidase. In EA.hy926 endothelial cells, oxidative stress caused either by incubating cells with xanthine oxidase together with hypoxanthine or with tert-butyl hydroperoxide leads to apoptosis, which is blocked when cells are allowed to adhere to vitronectin or fibronectin. The latter effect is inhibited by anti-αv and anti-α5 blocking monoclonal antibodies, indicating that the activation of integrins antagonizes the ROS-induced apoptosis [52]. In cardiomyocytes, oxidative stress induced by serum starvation leads to a rise in cytosolic cytochrome *c* levels and increased nuclear fragmentation. These effects are potentiated in cells with depleted β1 integrins while being reduced in cardiomyocytes treated with endothelin-1, which enhances β1 integrin expression [53]. Furthermore, integrins are also involved in protecting cells from ferroptosis defined as an iron-dependent form of programmed cell death with high levels of lipid ROS damaging the plasma membrane by the peroxidation of polyunsaturated fatty acids. In non-tumorigenic human mammary epithelial MCF-10A cells, the loss of α6β4 integrins increases susceptibility to ferroptosis induced by erastin, a cystine transporter inhibitor and ferroptosis inducer. The detachment of these cells triggers ferroptosis even in the absence of erastin, and this effect is potentiated in cells lacking α6β4 integrins. It has been found that α6β4 integrins protect cells from ferroptosis by suppressing the expression of acyl-CoA synthetase long-chain family member 4 (ACSL4), which produces polyunsaturated fatty acids that are primary targets of lipid peroxidation. ASCL4 expression is suppressed via the α6β4 integrin-induced Src-mediated phosphorylation of STAT3 that binds to the promoter and coding regions of the *ACSL4* gene [54].

### 4.2. Cadherins

E-cadherins increase the survival of many cancer cell types by reducing ROS levels. MMTV-PyMT invasive ductal carcinoma cells retain E-cadherin expression during growth, invasion, dissemination, and metastatic colonization. In these cells, experimentally induced E-cadherin deficiency is associated with enhanced cell invasion but also with increased apoptotic cell death. The detachment of cells from MMTV-PyMT organoids leads to a rise in ROS levels. The latter is strongly potentiated in cells with the experimentally abolished expression of E-cadherin, indicating that E-cadherin limits ROS generation. E-cadherin loss reduces the ability of cancer cells to form colonies, an effect inhibited by the antioxidant N-acetyl-cysteine, indicating that E-cadherin promotes the survival of cancer cells by reducing oxidative stress [55]. The knock-down of E-cadherin increases ROS generation and apoptosis in human breast cancer SKBR3 and MDA-MB-453 cells [56] while its overexpression in human breast cancer MDA-MB-231 cells, normally expressing low levels of E-cadherin, reduces ROS levels and increases the survival of cells incubated with 100 µM hydrogen peroxide [57]. These effects are blocked by the knock-down of phosphoglycerate dehydrogenase, the first and rate-limiting enzyme in the serine synthesis pathway providing precursors for the synthesis of glutathione, a major antioxidant. The ratio of reduced to oxidized glutathione is significantly higher in E-cadherin-overexpressing cells than in cells with low E-cadherin levels, indicating that E-cadherin mitigates oxidative stress by stimulating the serine synthesis pathway [57]. E-cadherin is also involved in protecting cancer cells from ferroptosis. In HepG2, PC9, H1650, and HCT116 epithelial cancer cells and patient-derived human malignant mesothelioma cells, interactions mediated by the extracellular domain of E-cadherin at cell-to-cell contacts trigger the suppression of ferroptosis induced by cystine starvation, erastin, and glutathione peroxidase 4 inhibitor RSL3. E-cadherin suppresses ferroptosis by activating the intracellular tumor suppressor NF2 (also known as merlin) and Hippo signaling pathway, leading to the inactivation of proto-oncogenic transcriptional co-activator YAP [58].

Other cadherins are also involved in protecting cells from oxidative-stress-triggered cell death. In patient-derived human malignant mesothelioma cells, N-cadherin suppresses ferroptosis by activating NF2 [58]. On the other hand, in human epithelial fibrosarcoma HT-1080 cells and non-small-cell lung cancer Calu-1 cells, the depletion of N-cadherin increases susceptibility to ferroptotic death by reducing membrane tension and favoring lipid peroxidation [59]. In human-umbilical-vein endothelial cells, serum deprivation leads to oxidative stress and apoptosis. T-cadherin overexpression increases the survival of these cells in the absence of serum through the activation of the PI3K/Akt/mTOR survival signaling pathway and concomitant suppression of the p38 MAPK proapoptotic pathway [60].

### 4.3. IgSF Members

The protective effects of this family members against ROS-related cell death have been particularly well studied for L1CAM, which is highly expressed in the nervous system. Single-chain variable fragment antibodies (scFvs) that bind to FnIII domains 1–3 of L1CAM improve the survival of human neuroblastoma SK-N-SH cells treated with 250 µM hydrogen peroxide. Interestingly, ScFvs that bind to Ig domains 1-4 of L1CAM do not reduce hydrogen-peroxide-triggered cell death. Furthermore, the levels of the anti-apoptotic protein Bcl-2 relative to the levels of the pro-apoptotic protein Bax are increased in hydrogen-peroxide-treated SK-N-SH cells co-treated with anti-FnIII1-3 scFvs, but not in cells co-treated with anti-Ig1-4 ScFvs [61], suggesting that L1CAM protects cells by regulating the expression of the genes involved in programmed cell death. The recombinant soluble extracellular domain of L1CAM reduces cell death in cultures of developing mouse cortical neurons incubated with 2 µM hydrogen peroxide for two hours [62], as well as in cultures of mouse cerebellar neurons incubated with 10 μM hydrogen peroxide for 24 h [47]. The latter effect is blocked in neurons expressing L1CAM with mutations inhibiting its cleavage and generation of the L1-70 fragment [47], which is imported into mitochondria [47] and nuclei [63], providing further evidence that L1CAM protects cells by regulating gene expression and possibly mitochondrial respiration. In human prostate cancer PC3 cells, L1CAM knock-down leads to a decrease in the expression of bcl-2 interacting protein 3 and different antioxidant enzymes that are important for ROS homeostasis including catalase, GPX1, GPX2, GPX4, GRP78, NOX5, SOD1, SOD2, and BIRC5 [64]. L1CAM knock-down also increases the sensitivity of PC3 cells to ionizing radiation, resulting in higher levels of apoptosis [64]. Interestingly, soluble L1CAM purified from the rat brain has significant intrinsic antioxidant activity since it reduces Fe^3+^ to Fe^2+^ to a degree similar to that of the known antioxidants melatonin and ascorbic acid [65]. Surface-immobilized L1CAM and soluble L1CAM reduce superoxide levels in rat microglial cells stimulated with phorbol-12-myristate-13-acetate, possibly by reducing oxidative species directly via intrinsic antioxidant activity [65]. Further research is needed to study the role that other members of this large family play in protecting cells from oxidative stress.

## 5. ROS Affect Cell Adhesion

### 5.1. Integrins

Oxygen free radicals and non-radical oxidants are important mediators of cellular injury in a number of pathophysiological processes including ischemia–reperfusion, atherosclerosis, and inflammation. Endothelial oxidant generation is followed by the recruitment and infiltration of leukocytes into the affected tissue. Integrins mediate the attachment of leukocytes to endothelial cells by binding to endothelial CAMs, mostly belonging to the IgSF family (discussed below). Earlier studies have shown that the integrin-dependent adhesion of inflammatory cells to endothelial cells or even plastic is increased by exposure to hydrogen peroxide, mimicking the inflammatory environment and inducing changes in integrin expression (Table 1). The adhesion of U-937 cells to plastic is strongly potentiated by increasing concentrations of hydrogen peroxide, with the maximum effect observed at 100 µM and then declining at 300 µM. This effect is blocked by antibodies against β2 integrin (also known as CD18) and α_M_ integrin (also known as CD11b), but not by antibodies against α_L_ or α_X_ integrins (also known as CD11a and CD11c), and depends on extracellular Mg^2+^, essential for the binding of CD11b/CD18 to its ligands [66]. In polymorphonuclear leukocytes, hydrogen peroxide induces a concentration-dependent increase in cell surface levels of α_M_ and β2 integrins observed within minutes at hydrogen peroxide concentrations starting at 0.1 mM and 0.5 mM, respectively. Hydrogen peroxide increases the attachment of polymorphonuclear leukocytes to the human aortic endothelial cells, and this effect is blocked by antibodies against β2 integrin [67]. The attachment of neutrophils to immobilized gelatin or fibrinogen is also potentiated in a dose-dependent manner by hydrogen peroxide (10 µM–100 mM) [68]. This effect is inhibited by antibodies against α_M_ integrins and requires Mg^2+^ (1 mM) as it is blocked by EDTA and does not take place in the presence of Ca^2+^ alone. The attachment of eosinophils to human umbilical vein epithelial cells is increased in a concentration-dependent manner when eosinophils are preincubated with 0.01–1 µM hydrogen peroxide while this effect is abolished at higher, 10 µM, hydrogen peroxide concentration. The hydrogen-peroxide-induced increase of eosinophil adhesion is accompanied by a rise in the expression of β2 and α_M_β2 integrins and is blocked by antibodies against β2 and α_M_β2 integrins but not by antibodies against α_L_β2 or α4 integrins. Hydrogen peroxide also increases the adhesion of eosinophils to immobilized recombinant human ICAM-1, a ligand for β2 integrin, but not to recombinant human vascular cell adhesion molecule-1 (VCAM-1), a ligand for α4 integrin [69].

ROS influence integrin-mediated adhesion in other cell types as well. The attachment of NIH-3T3 fibroblasts is severely impaired in fibroblasts pretreated with N-acetyl-cysteine or inhibitors of the Lox or Nox [14]. The long-term, 4-day-long, exposure of mouse mammary gland epithelial cells to 0.2 mM hydrogen peroxide, mimicking chronic inflammation, results in an increase in the expression of the α2, α3, α4, α5, α6, α7, β1, and β3 integrins while downregulating the expression of α1 integrin [70]. In neonatal cardiomyocytes, the Rac1-Nox-dependent generation of ROS in response to endothelin-1 enhances β1-integrin expression, both at mRNA and protein levels, via mitogen activated protein kinase (MEK)/ERK and EGFR-PI3K/Akt activation [53]. Promoters of at least some integrins are activated by NF-κB. For example, NF-κB binds to and activates β1 and β3 integrin promoters in breast cancer cells [71,72] and the αX integrin promoter in leukocytes [73]. The expression of integrins is reduced in osteosarcoma cells with inhibited NF-κB activity [74], suggesting that this redox-sensitive transcription factor may also be involved in the ROS-dependent upregulation of integrin expression. However, the exact mechanisms of the ROS-dependent integrin expression upregulation need to be further investigated.

In some cells, oxidative stress reduces the levels of integrins or affects their function. The attachment of mononuclear blood cells to collagen is inhibited when cells are pretreated with 0.2% hydrogen peroxide for 10 min. This effect can be reversed by 2.5 mM reduced glutathione [75]. The adhesion of human trabecular meshwork eye tissue cells to fibronectin, laminin, and collagen types I and IV is reduced when cells are treated with 1 mM hydrogen peroxide for 10 or 30 min. Interestingly, the levels of α5β1, αvβ3, and β1 integrins are not altered in these cells [76]. Oxidative stress has a bimodal effect on the adhesion of EA.hy926 endothelial cells to vitronectin and fibronectin. The induction of oxidative stress by preincubating cells with low concentrations of xanthine oxidase together with hypoxanthine or using chemical oxidant t-BHP promotes cell adhesion to vitronectin and fibronectin while higher xanthine oxidase and t-BHP concentrations decrease cell adhesion to these ECM components [52]. Low concentrations of xanthine oxidase and t-BHP increase levels of the αv integrin subunit, and decrease levels of α5 integrin, while not affecting the levels of β1 and β3 integrins [52]. In breast cancer MDA-MB-231 and SKBR3 cells, the induction of ROS using penfluridol results in the downregulation of expression of the α6, α5, β1, and β4 integrins. The expression of these integrins is regulated by Sp transcription factors. Penfluridol triggers the epigenetic downregulation of cMyc and cMyc-regulated miRNAs (miR27a and miR20a/miR17) and induction of the miR-regulated Sp transcriptional repressors ZBTB10 and ZBTB4, leading to the downregulation of Sp transcription factors Sp1, Sp3, and Sp4 [77]. Many non-surgical tumor treatment modalities such as ionizing radiation, chemotherapy using alkylating agents, or hyperthermia trigger ROS formation, which in turn is responsible for therapeutic cell damage. Hyperthermia causes a marked generation of ROS, and this effect is potentiated when hypothermia is combined with xanthine oxidase application and animals are allowed to breathe pure oxygen. In rat DS sarcoma cells, the expression of αv- and β3-integrins and αvβ3 dimers, but not β5 integrins, is reduced within 24 h after hypothermia-related oxidative stress, and this effect is inhibited by antioxidant vitamin E [78].

ROS modulate integrin-mediated adhesion not only by influencing integrin expression but also by changing their maturation, conformation, and interactions with other proteins. In astrocytes, amyloid β peptide-induced ROS production promotes the glycosylation-dependent conversion of β1 integrin precursor (105 kDa) into β1 integrin mature form (125 kDa) while this effect is inhibited by the Nox inhibitor [28]. Hydrogen peroxide acts on neutrophil adhesion through an intracellular signaling pathway involving tyrosine kinases and induces not only an increase in α_M_β2 integrin levels but also integrin activation as reflected by detection with conformation-specific antibodies [68]. Integrin-mediated leukocyte adhesion to ICAM-1 is increased by the ligation of toll-like receptors TLR2 and TLR5, which rapidly activate β2 integrins by inducing Rac1-dependent Nox2-mediated ROS production. The latter leads to the Rap1-GTPase-mediated increase in the intermediate- and high-affinity conformations of β2-integrins detected using conformation-specific antibodies [79]. Aortic smooth muscle cells form small α7β1 integrin-containing protrusions with increased redox potential and protein oxidation depending on the activity of Nox4. Hydrogen peroxide induces the oxidation of two cysteine residues within the extracellular domain of α7 integrin, thereby unlocking a disulfide bridge. The hydrogen peroxide treatment of α7β1 integrin in concentrations of up to 100 μM increases integrin binding activity to laminin [80]. This physiological redox regulation of α7β1 integrin is controlled by oxidoreductase thioredoxin-1, which cleaves the disulfide bridge and reverts the integrin into its lower activity conformation [81]. In HeLa cells, the oxidation of 37 kDa laminin receptor precursor (37LRP, also known as small ribosomal subunit protein uS2) by hydrogen peroxide leads to the formation of a disulfide bond between Cys148 and Cys163 of 37LRP, triggering changes in the protein conformation and leading to the association of 37LRP with β1 integrin and increased β1-integrin-mediated adhesion to laminin [82].

### 5.2. Cadherins

E-cadherin plays an important role in epithelial mesenchymal transition when cells switch from the polarized immotile epithelial type characterized by high E-cadherin levels to the motile mesenchymal type defined by low E-cadherin levels. The loss of E-cadherin is a key step in epithelial to mesenchymal transition and cancer progression that is associated with ROS production in various cancer cell types. For example, in rat DS sarcoma cells, hyperthermia-caused oxidative stress reduces the expression of E-cadherin but not N-cadherin. This effect can be inhibited with vitamin E [78]. In human hepatoblastoma HepG2 cells, pancreatic carcinoma PANC-1 cells, colon carcinoma HT-29 cells, and breast carcinoma MCF-7 cells, hypoxia-induced ROS generation results in the downregulation of E-cadherin expression and an increase in the expression of N-cadherin. E-cadherin expression is suppressed by SNAIL and β-catenin translocating to the nucleus in response to elevated ROS levels. These two proteins are regulated by glycogen synthase kinase 3β (GSK-3β), which is phosphorylated and inhibited in hypoxic cells in an MEK- and PI3K-dependent manner. Hypoxia-induced GSK3β phosphorylation and inactivation is reduced by inhibitors of mitochondrial ROS production, rotenone, or diphenyl-phenylene iodonium and can be mimicked by 50 µM hydrogen peroxide [83]. In addition, E-cadherin expression is regulated by hypoxia-inducible factor-1 (HIF-1). In ovarian cancer cells, ROS accumulation triggers a rise in the expression of HIF-1α and subsequent transcriptional induction of lysyl oxidase, which interacts with and increases the activity of SNAIL and represses E-cadherin expression [84]. In MCF-7 and MDA-MB-231 breast cancer cells, 40 µM hydrogen peroxide induces a ROS-dependent aberrant methylation of E-cadherin promoter, downregulating E-cadherin expression, at both mRNA and protein levels. This effect is also accompanied by SNAIL and SLUG upregulation, which can be blocked by the ERK inhibitor [85].

ROS suppress E-cadherin expression also in some non-cancer cell types. For example, in bronchial epithelial BEAS-2B cells, nickel chloride induces Nox1 and mitochondrion-dependent ROS production, which triggers a reduction in E-cadherin levels by increasing the levels of HIF1α, SNAIL, and SLUG and by inducing a ROS-dependent aberrant methylation of the E-cadherin promoter [86]. However, in some cases, ROS affect E-cadherin function not by downregulating its levels but rather by inducing its removal from the cell-to-cell junctions. For example, the long-term, 4-day-long, exposure of mouse mammary gland epithelial cells to hydrogen peroxide, mimicking chronic inflammation, results in the redistribution of E-cadherin from cell-to-cell contacts to the cytoplasmic vesicles not affecting the total E-cadherin levels [70].

The mechanisms of ROS-dependent E-cadherin regulation are evolutionary conserved. For example, in *Drosophila*, ROS are required for normal function of E-cadherins but also suppress their expression. In this model organism, the knock-down of superoxide dismutase 2, which dismutates superoxide to hydrogen peroxide, and Jafrac peroxidasin, which degrades hydrogen peroxide, lead to a reduction in E-cadherin protein levels [87,88]. The loss of E-cadherins is also observed in *Drosophila* embryos incubated with 10 mM hydrogen peroxide for 2 h [88], indicating that ROS suppress the expression of these proteins. Interestingly, the inhibition of Nox in *Drosophila* embryos causes a drop in ROS formation leading to the reduced E-cadherin localization at the enveloping layer cell junctions, an effect that can be blocked by 1 mM hydrogen peroxide. Nox inhibition reduces the capacity of disassociated blastoderm cells to aggregate but does not influence the total E-cadherin levels [89].

ROS also affect the levels and function of N-cadherins. In retinal pigment epithelial ARPE-19 cells, 200 µM hydrogen peroxide induces transient cell-to-cell disassociation via the inhibition of protein tyrosine phosphatases and activation of Src kinase, which phosphorylates p120-catenin and triggers the translocation of p120-catenin together with the internalization of N-cadherin from the cell–cell adhesion sites to an early endosomal compartment. Hydrogen peroxide does not significantly affect N-cadherin levels in these cells [90]. In contrast, in MDA-MB-468 breast cancer cells, hypoxia induces an increase in ROS levels accompanied by a rise in the levels of N-cadherin mRNA. The latter can also be induced by 1 mM hydrogen peroxide, the effect being inhibited by N-acetyl-cysteine [91].

Vascular endothelial (VE) cadherin plays an important role in maintaining the integrity and impermeability of endothelium. In endothelial cells from primary human umbilical veins, VE-cadherin-mediated cell–cell adhesion is lost when cells are loaded with a recombinantly produced cell-penetrating, constitutively active form of Rac. This effect occurs in parallel to and is dependent upon the intracellular production of ROS because it can be blocked by N-acetyl-cysteine and can be mimicked by incubating cells with 1 mM hydrogen peroxide [92]. ROS generation in human umbilical vein endothelial cells can also be induced by TNF, which triggers the tyrosine phosphorylation of VE-cadherin and its loss from adherens junctions, leading to intercellular gap formation and endothelial barrier dysfunction. These effects can be blocked by antioxidants and the inhibition of Nox and rely on the ROS-dependent activation of JNK [93]. Vascular endothelial growth factor (VEGF) enhances microvascular permeability through the disruption of the VE-cadherin-mediated adhesion caused by ROS. In human microvascular endothelial cells, VEGF induces both a Rac-1-dependent increase in ROS levels as well as the ROS-dependent tyrosine phosphorylation of VE-cadherin and β-catenin, disrupting the VE-cadherin/β-catenin complex and thereby controlling VE-cadherin levels at cell contacts [94]. In the brain, TNFα and IL-6 enhance ROS generation in microvascular endothelial cells by increasing the expression and co-association of membrane-bound gp91 and cytosolic p47 subunits of the Nox complex required for the complex activation. This rise in ROS levels leads to a drop in the levels of VE-cadherin and other components of cell junctions, resulting in an increase in endothelial cell layer permeability, an effect that can be abolished by ROS depleting agents or by blocking Nox [95].

Among other cadherins affected by ROS are T-cadherin and Fat1. In human umbilical vein endothelial cells, T-cadherin levels rise in response to 4 h long exposure to 1 mM hydrogen peroxide or oxidative stress caused by serum withdrawal, and these effects are suppressed by N-acetyl-cysteine. The increase in T-cadherin levels is inhibited by diphenyleneiodonium, an inhibitor of flavin-containing oxidases, among them Nox and xanthine oxidase, but not by rotenone, antimycin A, or *N*-monomethyl-L-arginine, a nitric oxide synthase inhibitor [60]. In vascular smooth muscle cells, mRNA and protein levels of Fat1 and the cell surface targeting of Fat1 are enhanced in response to angiotensin II-induced ROS production. Angiotensin II triggers ROS formation by enhancing the expression of Nox1, and the angiotensin-II-induced increase in Fat1 expression is blocked by the inhibitors of Nox and angiotensin II receptor blocker [96].

### 5.3. IgSF Members

ROS are involved in regulating the levels of multiple members of the IgSF both in the physiological and pathological contexts. In response to various inflammatory stimuli, endothelial cells recruit different types of leukocytes such as monocytes, lymphocytes, or neutrophils. They do this by selectively expressing different CAMs, such as VCAM-1, ICAM-1, or E-selectin (discussed below), which interact with CAMs expressed on the surface of immune cells, such as α4β1 integrin; very late activation antigen-4, which binds to VCAM-1; and macrophage αMβ2 integrin (also known as Mac-1), which binds to ICAM-1. The expression of CAMs is controlled by cytokines IL-1β and TNFα, derived from both inflammatory and endothelial cells, and can also be triggered by bacterial endotoxin lipopolysaccharide, the synthetic double-stranded RNA, polyinosinic:polycitidylic acid, and hydrogen peroxide, inducing the activation of NF-κB. The expression of CAMs can be specifically inhibited by antioxidants such as N-acetyl-cysteine and pyrrolidine dithiocarbamate. For example, in human umbilical vein endothelial cells, both lipopolysaccharides as well as cytokines IL-1β and TNFα induce the activation of NF-κB and transcriptional activation of the VCAM-1 promoter, and trigger an increase in expression of VCAM-1 and its cell surface levels, and these effects are inhibited by the pyrrolidine dithiocarbamate antioxidant [97]. Interestingly, the IL-1β-, TNFα- and lipopolysaccharide-induced expression of ICAM-1 and E-selectin are not significantly affected by pyrrolidine dithiocarbamate [97]. The exposure of human umbilical vein endothelial cells to anoxia followed by reoxygenation, designed to mimic ischemia–reperfusion-induced tissue injury, causes enhanced ROS production. A similar effect is induced by incubating cells with antimycin A. An elevation in ROS levels leads to an increase in the expression of ICAM-1 at the cell surface and enhanced adhesion of neutrophils to these cells, an effect that is blocked by antibodies against ICAM-1 [18]. In human aortic and microvascular endothelial cells, TNFα increases VCAM-1, ICAM-1, and E-selectin mRNA and cell surface protein levels. These effects can be blocked by the overexpression of dominant negative Rac-1, which does not inhibit TNFα-induced nuclear NF-κB binding activity or the inhibitor of NF-κB degradation but rather suppresses NF-κB-mediated transactivation. The induction of CAM expression is also blocked in cells overexpressing superoxide dismutase, which converts superoxide into hydrogen peroxide, but not in cells overexpressing catalase, which converts hydrogen peroxide to water, indicating that superoxide is a major regulator of CAM expression in these cells [98]. In primary bovine retinal endothelial cells and mouse capillary bEnd.3 endothelial cells, ICAM-1 levels are elevated in response to the application of VEGF at 100 ng/mL for 6 h. This effect is abolished by an inhibitor of PI3K and MnTMPyP, a cell-permeant mimic of superoxide dismutase, showing that VEGF induces an increase in ICAM-1 levels via the VEGF-receptor-mediated activation of PI3K and ROS generation [99]. The exposure of porcine aortic endothelial cells to 50 µM hydrogen peroxide induces a progressive increase in VCAM-1 expression [100]. Interestingly, hydrogen peroxide does not influence VCAM-1 expression in human umbilical vein and aortic endothelial cells. The hydrogen-peroxide-triggered VCAM-1 expression in porcine aortic endothelial cells is blocked by an inhibitor of IKK, a kinase regulating the activation of NF-κB, indicating that VCAM-1 expression is influenced via NF-κB-induced transcription. Antibodies against VCAM-1 block the hydrogen-peroxide-induced adhesion of human promonocytic U937 leukocytes to porcine aortic endothelial cells, showing that this interaction is mediated primarily by VCAM-1 [100]. Adipocytokine visfatin is a vascular inflammatory molecule that also increases the adhesion of leukocytes to endothelial cells by enhancing the expression of the inflammatory CAMs, ICAM-1 and VCAM-1. In human vascular and primary human umbilical vein endothelial cells, visfatin induces ROS production via the PI3K- and MAPK-dependent activation of Nox [101,102]. Visfatin triggers a rise in the levels of ICAM-1 and VCAM-1. This effect is inhibited by the inhibitor of Nox, knock-down of Nox4 expression, and inhibitors of IKK/NF-κB signaling, indicating that visfatin-induced ROS generation triggers an increase in ICAM-1 and VCAM-1 levels via IKK/NF-κB-induced gene expression [101,102].

ROS also regulate levels of CAMs in other cell types. In developing cultured mouse cortical neurons, exposure to hydrogen peroxide at the concentration 2 µM or higher induces a strong decrease in L1CAM levels [62]. This effect is reduced in cultures treated with the recombinant soluble extracellular domain of L1CAM [62], suggesting that homophilic interactions of L1CAM reduce the degradation of this molecule or upregulate its synthesis. Interestingly, oxidative stress triggered in human prostate cancer PC3 cells by hydrogen peroxide at the concentration of 10 mM results in an increase in L1CAM levels [64]. ROS production in PC3 cells caused by ionizing radiation and transforming growth factor β also increases L1CAM levels [64]. In cultured 5-day-old rat cortical neurons, oxidative stress caused by hydrogen peroxide at the concentration of 100 µM induces a gradual decrease in levels of full-length NCAM180 accompanied by a rise in levels of the 65 kDa cleavage product of NCAM. Hydrogen peroxide triggers an increase in levels of the matrix metalloproteinase-9, and the hydrogen-peroxide-induced cleavage of NCAM180 is blocked while cell death is reduced by an inhibitor of the matrix metalloproteinase-9, indicating that hydrogen peroxide influences NCAM180 cleavage by activating this protease [103]. Sodium fluoride applied at 40 mg/l and 80 mg/l for 24 h induces a rise in ROS levels in cultured seven-day-old rat primary hippocampal neurons. This effect is accompanied by a decrease in NCAM expression, at both mRNA and protein levels, and increased apoptosis [104].

The generation of ROS also influences IgSF CAMs indirectly by affecting enzymes that modify them. ROS induce the formation of carbonyl derivatives by the ROS-mediated oxidation of sidechains of histidine, arginine, and lysine into ketone or aldehyde derivatives. Glucuronyltransferase is an enzyme involved in the biosynthesis of HNK-1, a neural-specific carbohydrate epitope expressed on CAMs [105]. In primary cultured 7-day-old cortical neurons, an increase in ROS levels caused by the application of 25 µM amyloid β peptide leads to the carbonylation of glucuronyltransferase accompanied by a reduction in the levels of HNK-1-carrying NCAM, but does not affect the total levels of NCAM [106].

### 5.4. Selectins

Leukocyte–endothelial cell adhesion also involves selectins, which are also regulated by ROS. L-selectin is a carbohydrate-binding CAM that is constitutively expressed on the surfaces of non-activated granulocytes being shed after their activation. It is involved in the process of polymorphonuclear leukocyte rolling on the endothelial surface under conditions of shear stress. The exposure of these cells to increasing concentrations of hydrogen peroxide results in a concentration-dependent loss of L-selectin from the cell surface with a significant effect observed at 0.1 mM of hydrogen peroxide and the complete loss of L-selectin at 10 mM hydrogen peroxide. L-selectin mediates the early adhesion of polymorphonuclear leukocytes to stimulated endothelial cells under flow conditions, and this loss of L-selectins paralleled by an increase in integrin levels serves as a mechanism triggering integrin-dependent transendothelial migration [67].

The loss of L-selectins occurs via their shedding from the cell surface. In Jurkat T-cells, phorbol-myristate-acetate-induced ROS generation induces shedding of L-selectin, which depends on the activity of the TNFα-converting enzyme, also called ADAM17. Hydrogen peroxide activates ADAM17 presumably by an oxidative attack of the pro-domain thiol group and disruption of its inhibitory coordination with the Zn^2+^ in the catalytic domain of ADAM17 [107]. The latter is activated by extracellular ROS generated in leukocytes. Neutrophils are the most abundant leukocytes that eliminate intruders such as bacteria, fungi, or viruses. In human neutrophils, treatment with hydrogen peroxide or an increase in ROS levels induced, for example, by interleukin 8 (CXCL8) or various non-steroidal anti-inflammatory drugs, including flufenamic acid, meclofenamic acid, diclofenac, indomethacin, nimesulide, flurbiprofen, meloxicam, phenylbutazone, piroxicam, ketoprofen, and aspirin, results in a reduction in L-selectin expression at the cell surface. The latter depends on the extracellular superoxide anion-induced activity of ADAM17, which cleaves L-selectin. Accordingly, the downregulation of L-selectin expression can be blocked by inhibiting Nox [108,109]. L-selectin levels are also controlled by mitochondrial ROS. In naive CD4(+) T lymphocytes, 100 µM hydrogen peroxide induces the loss of L-selectins. ATP causes a similar effect by binding to P2X purinoceptor 7, which triggers ROS production in mitochondria. The effect of ATP can be augmented by enhancing mitochondrial ROS formation with rotenone or antimycin A. Interestingly, the Nox inhibitor apocynin does not affect the ATP-induced L-selectin loss [110]. In leukocytes, ROS generation is regulated by Rho. In murine neutrophils, the inhibition of Rho signaling leads to a significant drop in ROS levels and L-selectin shedding induced by bacterial-peptide fMLP, phorbol myristate acetate, and bacterial lipopolysaccharide [111].

In contrast to L-selectin in leukocytes, ROS enhance the expression of P- and E-selectins in endothelial cells. In endothelial cells of lung venular capillaries, ROS production caused by the elevation of lung capillary pressure or TNFα leads to increased P-selectin expression, which is blocked by rotenone and mitochondrial inhibitor carbonyl cyanide *p*-(trifluoro methoxy) phenylhydrazone, indicating that it depends on mitochondria as a ROS source [112,113]. In human saphenous vein endothelial cells, a sustained high stretch induces ROS generation and an increase in the cell surface levels of VCAM1 and E-selectin, which can be blocked by N-acetyl-cysteine [114]. In addition to affecting ICAM-1 expression, ROS production in human umbilical vein endothelial cells induced by various signals, including angiotensin II, thrombin, anoxia followed by reoxygenation, or antimycin A, also stimulates the cell surface expression of P- and E-selectin [18,115,116]. Thrombin-stimulated P-selectin upregulation results from the mobilization of the morphologically characteristic P-selectin-containing intracellular Weibel–Palade bodies from their “resting” intracytoplasmic location to the cell surface. This effect can be inhibited by antioxidants and inhibitors of Nox or xanthine oxidase, but not by rotenone or antimycin A [115]. A ROS-induced increase in the adhesion of neutrophils to endothelial cells is blocked by antibodies against P- and E-selectins [18]. The inhibition of NF-κB and AP-1 transcription factors attenuates the antimycin A-induced neutrophil adhesion and E-selectin expression, indicating that ROS promote selectin expression also at the transcriptional level [18]. In human pulmonary artery endothelial cells, TNFα stimulates ROS production, which can be blocked by N-acetyl-cysteine. Rising ROS levels trigger the activation of NF-κB, leading to an increase in E-selectin promoter activation, a rise in E-selectin mRNA levels, and its cell surface expression [117]. The exposure of human umbilical vein endothelial cells to TNFα results in enhanced leukocyte transmigration through the endothelial monolayer and higher E-selectin expression, both at the mRNA and protein levels, caused by elevated ROS levels and the augmented phosphorylation of the redox protein p66Shc and stress kinase JNK1/2. The overexpression of p66Shc triggers a rise in E-selectin levels and ROS production and these effects are blocked by the Nox inhibitor and the mitochondrial complex I inhibitors rotenone and thenoyltrifluoroacetone [118].

**Table 1:** Examples of changes in CAM expression in response to an increase in ROS levels.

CAM Family	Members	Cell Type	Effect of ROS on CAM Expression	References
Integrins	α1	mammary gland epithelial	reduce	[70]
	α2	mammary gland epithelial	increase	[70]
	α3	mammary gland epithelial	increase	[70]
	α4	mammary gland epithelial	increase	[70]
		eosinophil	no change	[69]
	α5	mammary gland epithelial	increase	[70]
		EA.hy926 endothelial	reduce	[52]
		MDA-MB-231 and SKBR3 breast cancer	reduce	[77]
	α6	mammary gland epithelial	increase	[70]
		MDA-MB-231 and SKBR3 breast cancer	reduce	[77]
	α7	mammary gland epithelial	increase	[70]
	αL	U-937 histiocytic lymphoma	no change	[66]
	αM	polymorphonuclear leukocyte	increase	[67]
		eosinophil	increase	[69]
		U-937 histiocytic lymphoma	increase	[66]
	αV	EA.hy926 endothelial	increase	[52]
		DS sarcoma	reduce	[78]
	αX	U-937 histiocytic lymphoma	no change	[66]
	β1	MDA-MB-231 and SKBR3 breast cancer	reduce	[77]
		neonatal cardiomyocyte	increase	[53]
		EA.hy926 endothelial	no change	[52]
		mammary gland epithelial	increase	[70]
		trabecular meshwork eye	no change	[76]
	β2	polymorphonuclear leukocyte	increase	[67]
		eosinophil	increase	[69]
		U-937 histiocytic lymphoma	increase	[66]
	β3	EA.hy926 endothelial	no change	[52]
		mammary gland epithelial	increase	[70]
		DS sarcoma	reduce	[78]
	β4	MDA-MB-231 and SKBR3 breast cancer	reduce	[77]
	β5	DS sarcoma	no change	[78]
Cadherins	Fat1	vascular smooth muscle	increase	[96]
	E-cadherin	BEAS-2B bronchial epithelial	reduce	[86]
		*Drosophila* embryo	reduce	[88]
		HepG2 hepatoblastoma	reduce	[83]
		HT-29 colon carcinoma	reduce	[83]
		MCF-7, MDA-MB-231 breast carcinoma	reduce	[83,85]
		mammary gland epithelial	no change	[70]
		ovarian cancer	reduce	[84]
		PANC-1 pancreatic carcinoma	reduce	[83]
		DS sarcoma	reduce	[78]
	N-cadherin	ARPE-19 retinal pigment epithelial	no change	[90]
		HepG2 hepatoblastoma	increase	[83]
		HT-29 colon carcinoma	increase	[83]
		MCF-7, MDA-MB-468 breast carcinoma	increase	[83,91]
		PANC-1 pancreatic carcinoma	increase	[83]
		DS sarcoma	no change	[78]
	T-cadherin	umbilical vein endothelial	increase	[60]
	VE-cadherin	umbilical vein endothelial	reduce	[92,93]
		microvascular endothelial	reduce	[94,95]
IgSF	ICAM-1	aortic endothelial	increase	[98]
		microvascular endothelial	increase	[98]
		umbilical vein endothelial	increase	[18,97,101,102]
		retinal endothelial	increase	[99]
		capillary bEnd.3 endothelial	increase	[99]
		vascular endothelial	increase	[101,102]
	L1CAM	cortical neurons	decrease	[62]
		PC3 prostate cancer	increase	[64]
	NCAM	cortical neurons	decrease	[103]
		hippocampal neurons	decrease	[104]
	VCAM-1	aortic endothelial	increase, no change	[98,100]
		microvascular endothelial	increase	[98]
		umbilical vein endothelial	increase, no change	[97,100,101,102]
		vascular endothelial	increase	[101,102]
		saphenous vein endothelial	increase	[114]
Selectins	E-selectin	aortic endothelial	increase	[98]
		microvascular endothelial	increase	[98]
		umbilical vein endothelial	increase	[18,97,115,116,118]
		saphenous vein endothelial	increase	[114]
		pulmonary artery endothelial	increase	[117]
	L-selectin	Jurkat T-cells	reduce	[107]
		polymorphonuclear leukocytes	reduce	[67]
		neutrophils	reduce	[108,109,111]
		naive CD4(+) T lymphocytes	reduce	[110]
	P-selectin	lung venular capillaries endothelial	increase	[112,113]
		umbilical vein endothelial	increase	[18,115,116]

## 6. CAMs and ROS Interplay in Diseases

The reciprocal relationship between CAMs and ROS plays a significant role in various diseases. It is probably most well characterized in diseases linked to inflammation, covered in several recent reviews and discussed here only briefly. ROS play a key role in atherosclerosis [119], where a rise in ROS levels in endothelial cells drives an increase in the expression of CAMs such as E-selectin, P-selectin and VCAM-1 [120]. Increased levels of CAMs promote the attachment of the inflammatory cells to endothelium at the early stages of atherosclerotic lesion formation [120]. The inhibition of ROS production, for example, via the ablation of expression of different Nox or by reducing mitochondrial ROS production, leads to a reduction in the expression of CAMs in vascular walls and attenuates lesion formation [119,120]. Elevated ROS production by endothelial cells is also triggered during septic shock, a condition caused by a dysregulated host response to infection whereby the circulation cannot deliver adequate blood flow, ultimately leading to organ dysfunction [121]. During sepsis or acute inflammation, a rise in ROS levels upregulates the expression of CAMs in endothelial cells, enhancing the recruitment of phagocytic cells to infectious sites. The infiltration of activated leukocytes into the tissue contributes to tissue injury and ultimately organ dysfunction and failure [121,122].

Cancer cells produce high levels of ROS to maintain neoplastic state [123]. ROS play a key role in epithelial–mesenchymal transition, driving cancer cell metastasis and drug resistance [124,125]. Epithelial cells are laterally conjoined to form layers or polarized sheets. This organization is lost in mesenchymal cells, which rarely exhibit conjunctions with adjacent cells. E-cadherins play a key role in maintaining epithelial cell–cell junctions. Rising levels of ROS trigger the loss of E-cadherin and activate the expression of migration-promoting N-cadherin, a switch that promotes the epithelial–mesenchymal transition [126].

In the brain, ROS in the low physiological range of concentrations promote neuronal development [127] and maintain the function of mature neurons during learning and memory formation [128]. CAMs play an important role in regulating brain development and function [5] and are implicated in the pathogenesis of neurodevelopmental disorders [129]. The role of ROS in neurodevelopmental disorders associated with the loss of function of CAMs needs, however, to be determined.

In healthy cells, ROS production and changes in ROS levels are highly compartmentalized, being spatially and temporally controlled by cellular antioxidant systems [34,130]. The loss of this compartmentalization, resulting in an increase in ROS levels in multiple cellular compartments, leads to oxidative stress and ultimately cell death associated with various pathological conditions [34]. For example, ROS overproduction in the brain contributes to neuronal dysfunction and death in neurodegenerative diseases [131], which are linked to changes in levels and functions of neuronal CAMs [132]. Whether the ROS-triggered loss of neuronal CAMs, such as L1CAM or NCAM, contributes to neurodegenerative disorders by affecting the CAM-dependent regulation of ROS production and antioxidant systems is a topic for future research.

## 7. Conclusions and Future Directions

The current evidence indicates that different types of integrins and some members of other major families of CAMs induce a rise in cytosolic ROS levels in response to binding to their extracellular ligands. Several sources of ROS, including Lox, Nox, and mitochondria, are implicated in CAM-dependent ROS generation, but the exact mechanisms vary depending on the cell type and CAMs involved. The CAM-dependent increase in ROS levels is necessary for the oxidation-dependent modification of the cytosolic proteins including the cytoskeleton components and key enzymes involved in CAM-dependent signaling. Importantly, CAMs also protect cells from oxidative injury by activating the synthesis of antioxidants, suppressing molecular pathways triggering cell death and stimulating the expression of proteins reducing oxidative stress. It is tempting to speculate that CAM-dependent mechanisms providing protection against ROS are co-activated in parallel to CAM-dependent ROS production in order to prevent the self-inflicted damage by ROS. It should be noted, however, that the exact spatiotemporal relationship between the CAM-dependent ROS formation and their clearance has not been well established, and different CAMs may be involved in these mechanisms.

ROS themselves are involved in regulating the levels and function of CAMs by controlling their expression, conformation, degradation, and interactome. Interestingly, changes in the expression of integrins induced by rising ROS levels vary in different cell types (Table 1), suggesting that different cells have unique ROS-dependent mechanisms regulating the expression of these CAMs. While the effects of ROS on CAMs of other major families are more consistent (Table 1), L1CAM is a notable example of an IgSF CAM that is differently regulated by ROS in neurons and cancer cells. Further research is needed to understand whether many other CAMs are regulated by ROS in a cell-type- and tissue-specific manner and whether this regulation is altered in disease.

Although substantial progress has been made in our understanding of the reciprocal relationship between CAMs and ROS, many questions remain unanswered. In addition to integrins, CAMs belonging to other families, such as the IgSF member NCAM, interact with and regulate Rac activators [133]; however, their role in regulating ROS levels needs to be investigated. CAMs induce a plethora of intracellular signaling pathways, and whether ROS play a role in regulating many more enzymes in these pathways has yet to be discovered. Those enzymes known to be activated by ROS formed in response to integrin activation, such as Src kinase family members, are also activated by other CAMs, including IgSF family members of the L1CAM and NCAM families [134,135,136]. However, it remains to be determined whether ROS-dependent activation is a universal mechanism utilized by all CAMs. Increased ROS production is featured in many pathological conditions, notably in many neurodegenerative disorders [131,137]. Its effects on the expression and function of CAMs invite a thorough investigation. By answering these questions, future research may pave the way for new treatments of various conditions associated with high ROS levels and altered CAM function.

## Data Availability

No new data were created or analyzed in this study. Data sharing is not applicable to this article.

## Abbreviations

The following abbreviations have been used in this manuscript:

37LRP	37 kDa laminin receptor precursor
ACSL4	Acyl-CoA synthetase long-chain family member 4
CAMs	Cell adhesion molecules
CHL1	Close homologue of L1
CXCL8	Interleukin 8
ECM	Extracellular matrix
EGF	Epidermal growth factor
EGFR	Epidermal growth factor receptor
FAK	Focal adhesion kinase
GCL	Glutamate cysteine ligase
HIF-1	Hypoxia-inducible factor-1
ICAM-1	Intercellular cell adhesion molecule 1
Ig	Immunoglobulin
IgSF	Immunoglobulin superfamily
JNK	c-Jun N-terminal protein kinase
L1CAM	L1 cell adhesion molecule
Lox	Lipoxygenase
MAPK	Mitogen-activated protein kinase
NCAM	Neural cell adhesion molecule
Ndufv2	NADH dehydrogenase ubiquinone flavoprotein 2
NF-κB	Nuclear factor kappa B
Nox	NADPH oxidase
PI3K	Phosphatidylinositol 3-kinase
PKC	Protein kinase C
Rac-1	Ras-related C3 botulinum toxin substrate 1
RGDS	Tetrapeptide Arg–Gly–Asp–Ser
ROS	Reactive oxygen species
TMIGD1	Transmembrane and immunoglobulin domain-containing protein 1
TNFα	Tumor necrosis factor α
VCAM-1	Vascular cell adhesion molecule-1
VE-cadherin	Vascular endothelial cadherin
VEGF	Vascular endothelial growth factor
